# Standardizing cervical lymph node evaluation in papillary thyroid carcinoma: diagnostic accuracy of Node-RADS on ultrasound

**DOI:** 10.3389/fendo.2026.1709522

**Published:** 2026-01-28

**Authors:** Hongjun Zhang, Danni He, Muyi Mao, Renjie Li, Tinghui Yin, Bo-wen Zheng, Jiayi Zheng, Tao Wu, Jie Ren, Zuofeng Xu

**Affiliations:** 1 Seventh Affiliated Hospital, Sun Yat-sen University, Shenzhen, China; 2 Third Affiliated Hospital of Sun Yat-Sen University, Guangzhou, China; 3 Guangdong Women and Children Hospital, Guangzhou, China

**Keywords:** papillary thyroid carcinoma, cervical lymph node, ultrasonography, Node reporting and data system, risk stratification

## Abstract

**Objectives:**

To assess the diagnostic performance of the Node Reporting and Data System (Node-RADS) in ultrasonographic evaluation of cervical lymph node (LN) metastasis in papillary thyroid carcinoma (PTC) and compare it with conventional suspicious sonographic features.

**Materials and methods:**

This retrospective study included 227 PTC patients (269 LNs) who underwent preoperative cervical LN ultrasound and biopsy (September 2022–December 2023). Two radiologists independently assigned Node-RADS scores (1–5) based on LN size and morphologic features (shape, border, echogenicity). Diagnostic performance was evaluated using ROC analysis, with histopathology as the reference standard. Node-RADS was compared to five American Thyroid Association (ATA)-defined suspicious features (microcalcifications, cystic changes, etc.). Multivariable analysis assessed an integrated model combining Node-RADS with peripheral vascularity.

**Results:**

Metastatic LNs (63.9%, 172/269) exhibited higher Node-RADS scores (malignancy rates: 35.7% for score 1 vs. 88.2% for score 5). Node-RADS achieved an AUC of 0.720 (95% CI: 0.662–0.773), surpassing individual features (best AUC: 0.703 for peripheral vascularity). Inter-reader agreement was good (*κ* = 0.67). A combined Node-RADS and peripheral vascularity model significantly improved diagnostic performance (AUC: 0.825, *p* < 0.01).

**Conclusions:**

Node-RADS offers standardized, reproducible risk stratification for cervical LN metastasis in PTC, with moderate diagnostic accuracy. Integration with peripheral vascularity enhances performance, supporting its potential clinical utility in preoperative assessment.

## Introduction

Papillary thyroid carcinoma (PTC) comprises approximately 80–90% of thyroid cancers (1). Its global incidence has been rising steadily, largely due to the increased use of high-resolution imaging and fine-needle aspiration biopsy (FNAB) (2, 3). Although PTC generally exhibits indolent behavior, cervical lymph node (LN) metastasis occurs in up to 80% of patients and plays a critical role in staging, prognosis, and recurrence of PTC, however, accurate preoperative assessment remains challenging (4). Ultrasonography (US) is the primary imaging modality for evaluating thyroid nodules and cervical LNs, favored for its non-invasiveness, accessibility, and high spatial resolution (5, 6). However, its diagnostic accuracy in identifying metastatic lymph nodes in PTC patients remains suboptimal (7, 8). Current US-based evaluations depend critically on operator experience and interpretation of heterogeneous sonographic features, such as shape, margin, echogenicity, vascularity, and calcifications (1, 9). This variability underscores the need for a more objective and reproducible scoring system to improve the diagnostic performance of US in detecting cervical LN metastasis.

Structured imaging reporting systems have demonstrated clinical value in other malignancies. Systems such as the Breast Imaging Reporting and Data System (BI-RADS) and Prostate Imaging Reporting and Data System (PI-RADS) have improved diagnostic consistency by standardizing the interpretation of imaging findings (10–13). Despite the high prevalence of LN metastasis in PTC, a comparable standardized system for cervical LN evaluation on US remains unavailable for wide implementation.

To enhance objectivity and standardization in LN assessment, the Node Reporting and Data System (Node-RADS) 1.0 was introduced across CT and MR imaging modalities (14). Preliminary studies suggested its potential utility for LN assessment in various malignancies (15–19). However, its performance in US-based assessment of cervical LNs in PTC patients has not been systematically investigated.

This study aimed to investigate the diagnostic performance of Node-RADS for cervical LNs in PTC patients. We further compared its discriminative ability with individual suspicious sonographic features and explored an integrated diagnostic model to improve clinical applicability.

## Materials and methods

### Ethical approval and study design

This retrospective cohort study was conducted from the third affiliated hospital of Sun Yat-sen University between September 2022 and December 2023. It was approved by the institutional review board of our hospital, and informed consent was waived.

### Patient selection criteria

Consecutive patients were included based on the following criteria (1): histologically confirmed PTC (2); presence of cervical LNs suspected malignant on US (3); underwent both diagnostic US and subsequent US-guided FNA or excisional biopsy between September 2022 and December 2023 (4); availability of complete B-mode US and color Doppler flow imaging (CDFI) data.

Exclusion criteria comprised (1): inconclusive pathological results (2); non-diagnostic US image quality (3); prior radioactive iodine (¹³¹I) therapy (4); concurrent malignancies (5); missing clinical or imaging data.

**Figure 1:**
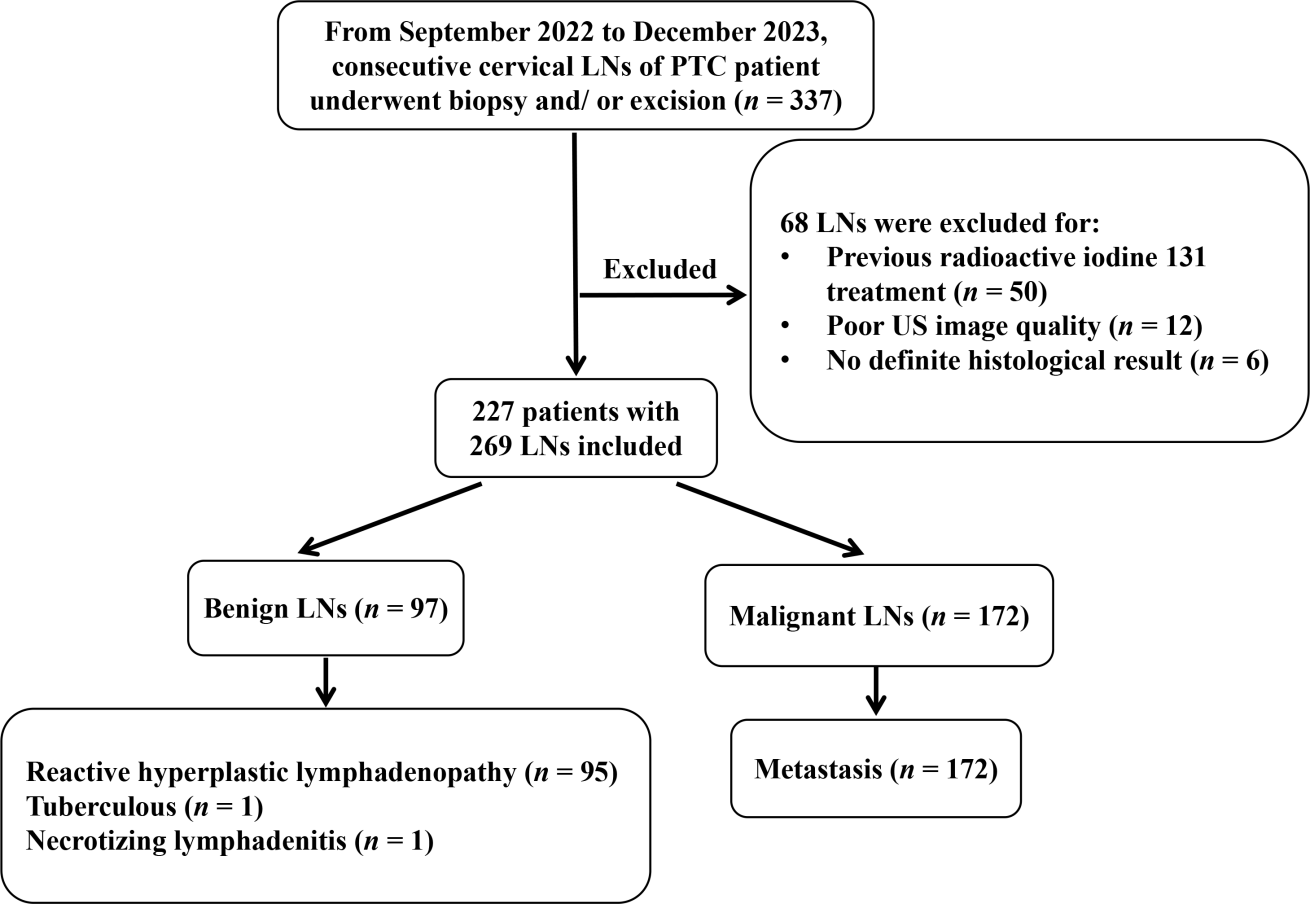
Flowchart for patient selection. LN, lymph node; PTC, papillary thyroid carcinoma; US, ultrasound.

The patient selection process is outlined in **Figure 1**. Histopathological analysis of US-guided and/or excision biopsies served as the reference standard, with benign LNs undergoing 6-month US follow-up (20, 21).

### Sample size calculation

A formal sample size calculation was performed before the analysis. For the development of the multivariable logistic regression model, the rule of thumb of 10 events per variable (EPV) was applied (22–24). With 6 predictor variables in the model and an anticipated event (malignancy) rate of approximately 40% based on prior literature in similar populations (1), a minimum of 150 samples (60 events/0.4) was required. Our final cohort of 269 lymph nodes, therefore, provides adequate statistical power for robust model development and validation.

### Ultrasound protocol

All US examinations and biopsies were performed using high-resolution linear transducers (7–15 MHz) under standardized protocols, including 5 standardized ultrasonography instruments: ALOKA Prosound Alpha 6 (Hitachi), ALOCA ARIETTA 60 (Hitachi), ALOCA ARIETTA 70 (Hitachi), GE Logiq E9 (GE), and Acuson Sequoia (Siemens). Three fellowship-trained radiologists (10–15 years experience in thyroid imaging) performed all scans and documented LN features, including anatomical location, short-axis diameter, short-to-long axis ratio, cortical thickness, internal echotexture (echogenicity, calcifications, cystic changes), and vascularity pattern (hilar, peripheral, or mixed). Baseline demographics (age, sex) and pathological results were recorded. In accordance with standard clinical practice, all lymph nodes subjected to biopsy were initially marked under ultrasound guidance. During subsequent surgical excision, the nodes were identified and matched based on their anatomical location and correlative skin markings.

### Node-RADS assessment

Before the study, two radiologists (with 12 and 4 years of experience, respectively) attended a dedicated training session on the Node-RADS criteria, which included a review of the scoring guidelines and a set of 20 practice cases (not included in the final analysis) to ensure consistent understanding and application of the criteria. In clinical practice, assigning a Node-RADS score requires approximately 1–2 minutes per case. To minimize potential bias, the readers were blinded to all clinical information, subsequent pathological results, and the individual assessments of their fellow readers throughout the Node-RADS scoring process. The Node-RADS score is determined by combining scores from LN size and configuration (including shape, border, and cortical echogenicity) (14). The size category was stratified as normal, enlarged, or bulky, while “configuration” was evaluated based on border regularity, shape, and cortical echogenicity. Each subcategory contributed a partial score, and the cumulative sum determined the final Node-RADS score, ranging from 1 to 5, indicating progressively increasing suspicion of malignancy.

LNs in cervical regions were categorized as “enlarged” if the short-axis diameter was ≥ 10 mm. Vascularity was assessed on CDFI and graded as absent (G0), minimal (G1; one or two discrete color signals < 1 mm), moderate (G2), or marked (G3; ≥4 discrete vessels) (25). Discrepancies in scoring were resolved by choosing the higher of the two scores.

### Statistical analyses

LNs were classified as benign or metastatic based on pathological confirmation. Continuous variables were expressed as mean ± standard deviation or median [interquartile range], and categorical variables as counts with percentages. Group comparisons used the independent *t*-test or Mann–Whitney U test for continuous variables, and the chi-square test for categorical variables.

Receiver operating characteristic (ROC) curves were constructed to evaluate the diagnostic performance of Node-RADS and compared to five American Thyroid Association (ATA)-defined suspicious sonographic features: focal hyperechogenicity, microcalcification, cystic degeneration, round shape, and peripheral vascularity (1). Sensitivity, specificity, positive predictive value (PPV), and negative predictive value (NPV) were calculated at the optimal Node-RADS cutoff, determined by the maximum Youden index.

Potential predictors were initially screened using univariable logistic regression. To avoid multicollinearity, the individual components of the Node-RADS score (e.g., size, shape, echogenicity) were not re-evaluated as separate variables. Candidate variables with a p-value of less than 0.10 from the univariable analysis were included in the subsequent multivariable model. A final multivariable logistic regression model was then constructed with the independent predictors. The discriminative performance of this final model was evaluated using ROC curve analysis. To mitigate the risk of overfitting and to substantiate the robustness of the combined diagnostic model, an internal validation was performed using the 10-fold cross-validation method. The average area under the receiver operating characteristic curve (AUC) derived from these validation cycles is reported. Inter-reader agreement for Node-RADS scoring was assessed using Cohen’s kappa statistics (*κ*), interpreted as follows: excellent (*κ* > 0.80), good (*κ* = 0.61–0.80), moderate (*κ* = 0.41–0.60), fair *(κ* = 0.21–0.40), and poor (*κ* < 0.20). In an effort to investigate a potential learning curve, a *post-hoc* analysis was performed by stratifying the reading process into first and second halves. All statistical analyses were performed using SPSS version 25.0, MedCalc version 20.0, and R software version 44.5.2, with a two-tailed *p*-value < 0.05 considered statistically significant.

## Result

### Patient characteristics

A total of 227 patients (mean age of 40 ± 14 years; age range, 10–82 years; 71.8% female) with 269 cervical LNs were included in the final analysis. Excluded cases comprised 12 with suboptimal image quality, 6 with inconclusive histology, and 50 with prior ^131^I therapy (**Figure 1**).

Pathological confirmation was obtained via FNA alone (*n* = 85, 31.6%), core-needle biopsy (CNB) alone (*n* = 37, 13.8%), FNA with excision (*n* = 115, 42.8%), and CNB with excision (*n* = 32, 11.9%) (**Table 1**). The high concordance rate of 92.2% (107/116) between FNA and subsequent surgical excision, which is consistent with the previous literature (26, 27), supports FNA as a robust diagnostic standard in our study. Final pathology revealed 172 metastatic LNs (63.9%) and 97 benign LNs (36.1%), including reactive hyperplasia (*n* = 95), necrotizing lymphadenitis (*n* = 1), and tuberculous lymphadenitis (*n* = 1).

**Table 1:** Baseline demographic and clinical characteristics of the study.

Characteristics	Value (*n* = 269)
Age (years)^*^	40 ± 14
Sex (female)^†^	163 (71.8)
Size (mm)^‡^	
Long-axis	11.6 (8.2, 16.4)
Short-axis	5.5 (4.0, 7.6)
Short-to-long axis ratio	
< 2	120 (44.6)
≥ 2	149 (55.4)
Shape	
Oval	145 (53.9)
Round	107 (39.8)
Irregular	17 (6.3)
Internal echotexture	
Focal hyperechogenicity	99 (36.8)
Microcalcifications	92 (34.2)
Cystic degeneration	34 (12.6)
Border	
Smooth	251 (93.3)
Irregular or ill-defined	18 (6.7)
Node-RADS	
1	28 (10.4)
2	55 (20.4)
3	46 (17.1)
4	64 (23.8)
5	76 (28.3)
Cortical thickness (mm)^‡^	5.0 (3.7, 5.0)
Vascular pattern	
Hilar	166 (61.7)
Peripheral	52 (19.3)
Mixed	51 (19.0)
Vascular quantity on CDFI	
G0	75 (27.9)
G1	110 (40.9)
G2	34 (12.6)
G3	50 (18.6)
Pathological diagnosis method	
Fine-needle aspiration	85 (31.5)
Core needle biopsy	37 (13.8)
Fine-needle aspiration and Excision	115 (42.8)
Core needle biopsy and Excision	32 (11.9)
Pathology	
Malignant	172 (63.9)
Benign	97 (36.1)

Unless otherwise indicated, data are number of lymph nodes; data in parentheses are percentages. ^*^Data are presented as mean ± standard deviation. ^†^Data are number of participants. ^‡^Expressed as median (interquartile ranges). Node-RADS, Node Reporting and Data System; CDFI, color Doppler flow imaging.

### Node-RADS score distribution and interreader agreement

**Figure 2:**
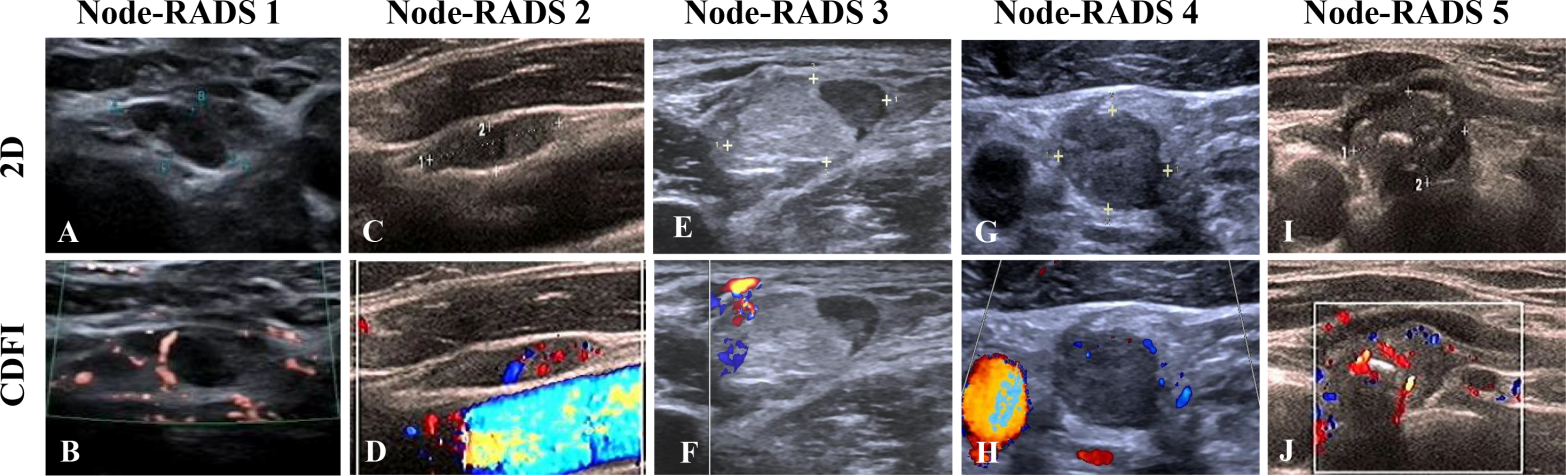
Representative cervical LN of different Node-RADS scores. **(A, B)** Node-RADS score 1: short axis = 4 mm (0 points), homogeneous texture (0 points), smooth borders (0 points), and kidney-bean-like shape with fatty hilum (0 points); **(C**, **D)** Node-RADS score 2: short axis = 5 mm (0 points), heterogeneous texture (1 point), smooth border (0 points), and kidney-bean-like shape without fatty hilum (0 points); **(E**, **F)** Node-RADS score 3: short axis = 10 mm (1 point), heterogeneous texture (1 point), smooth border (0 points), and oval shape without fatty hilum (0 points); **(G**, **H)** Node-RADS score 4: short axis = 9 mm (0 points), heterogeneous (1 point), irregular border (1 point), and spherical without fatty hilum (1 point); **(I**, **J)** Node-RADS score 5: short axis = 16 mm (1 point), calcification (3 points), irregular border (1 point), and spherical without fatty hilum (1 point). LN, lymph node; CDFI, Color Doppler flow imaging; Node-RADS, Node Reporting and Data System.

Among the 269 LNs, the distribution of Node-RADS scores was as follows: score 1 (*n* = 28), score 2 (*n* = 55), score 3 (*n* = 46), score 4 (*n* = 64), and score 5 (*n* = 76). The corresponding malignancy rates were 35.7%, 50.9%, 45.7%, 71.9%, and 88.2%, respectively. Representative ultrasound images of each Node-RADS category are presented in **Figure 2**.

Inter-reader diagnostic consistency for Node-RADS scoring yielded a Cohen’s *κ* of 0.67 (95% confidence interval [CI]: 0.60–0.73), indicating good concordance between the two radiologists.

The inter-reader agreement was found to be κ = 0.66 in the first half and κ = 0.68 in the second half. While this indicates a trend towards improved concordance, the difference was not statistically significant (P = 0.77). This finding may suggest a rapid familiarization period with the Node-RADS system.

### Diagnostic performance of Node-RADS and five suspected US features

Node-RADS demonstrated superior diagnostic performance over individual sonographic features, achieving an area under the curve (AUC) of 0.720 (95% CI: 0.662–0.773, *p* < 0.001). The optimal cutoff score was > 3, yielding a sensitivity of 65.7% (95% CI: 58.1% - 72.8%) and a specificity of 72.2% (95% CI: 62.1% - 80.8%). The positive predictive value (PPV) and negative predictive value (NPV) were 80.7% (95% CI: 74.9% - 85.4%) and 54.3% (95% CI: 48.3% - 60.2%), respectively (**Table 2**).

**Table 2:** Diagnostic performance for detecting cervical lymph node metastasis.

Characteristics	AUC (95% CI)	*P*	Sensitivity (95% CI)	Specificity (95% CI)	PPV (95% CI)	NPV (95% CI)
Node-RADS	0.720 (0.662 - 0.773)	< 0.01	65.7% (58.1% - 72.8%)	72.2% (62.1% - 80.8%)	80.7% (74.9% - 85.4%)	54.3% (48.3% - 60.2%)
Focal hyperechogenicity	0.578 (0.517 - 0.638)	0.01	42.4% (35.0% - 50.2%)	73.2% (63.2% - 81.7%)	73.7% (65.9% - 80.3%)	41.8% (37.6% - 46.1%)
Microcalcification	0.663 (0.603 - 0.719)	< 0.01	45.9% (38.3% - 53.7%)	86.6% (78.2% - 92.7%)	85.9% (78.1% - 91.2%)	47.5% (43.5% - 51.4%)
Degeneration	0.542 (0.481 - 0.603)	0.03	15.7% (10.6% - 22.0%)	92.8% (85.7% - 97.0%)	79.4% (63.6% - 89.5%)	35.3% (30.3% - 40.3%)
Round Shape	0.585 (0.524 - 0.645)	0.01	51.7% (44.0% - 59.4%)	63.9% (53.5% - 73.4%)	71.8% (65.3% - 77.5%)	42.8% (37.6% - 48.1%)
Peripheral vascularization	0.703 (0.644 - 0.757)	< 0.01	53.5% (45.7% - 61.1%)	88.7% (80.6% - 94.2%)	89.3% (82.5% - 93.7%)	51.8% (47.4% - 56.2%)

AUC, area under the curve; CI, confidence interval; PPV, positive predictive value; NPV, negative predictive value; Node-RADS, Node Reporting and Data System.

**Figure 3:**
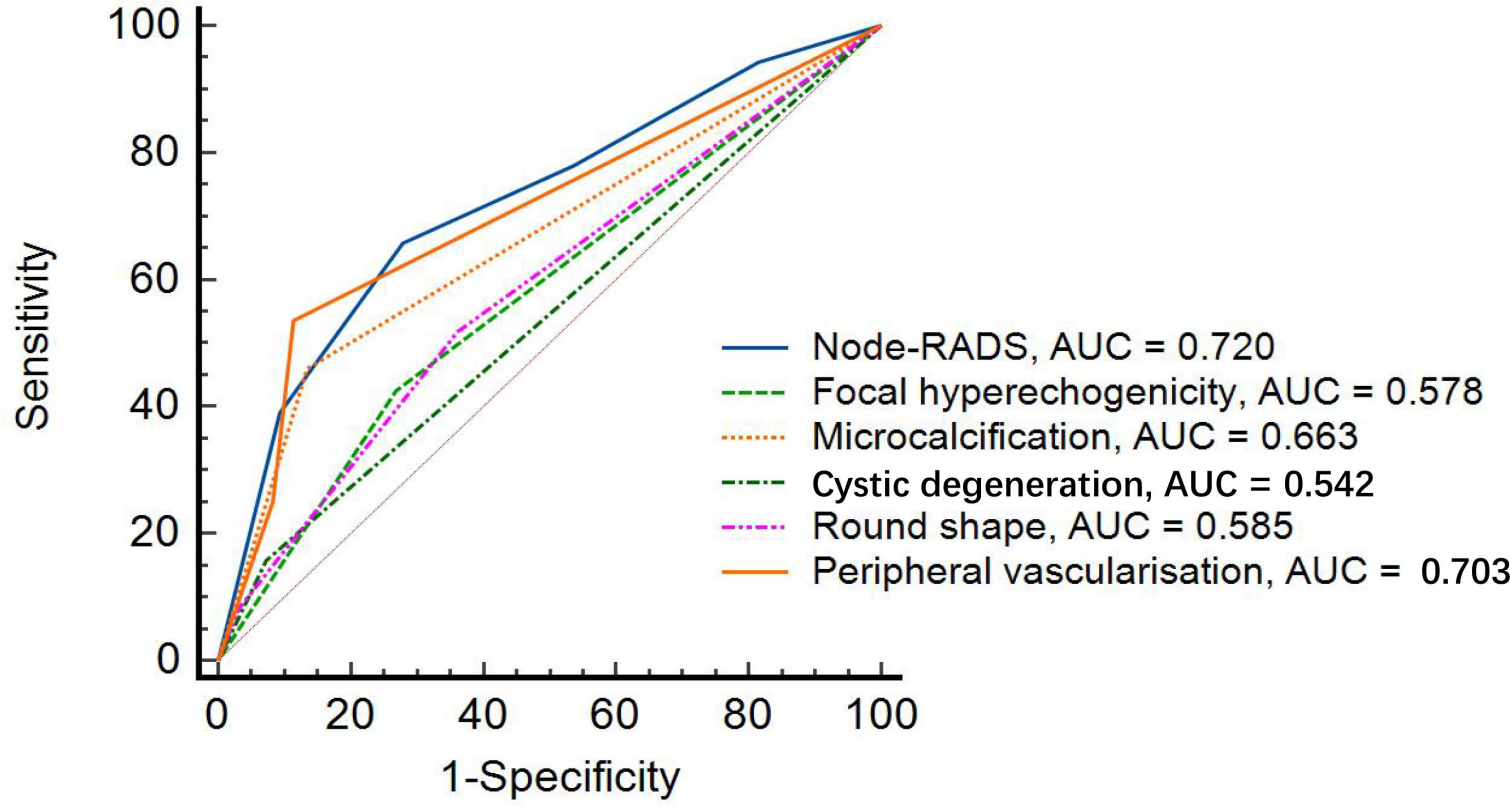
Diagnosis performance of Node-RADS and five suspected US features. Node-RADS, Node Reporting and Data System; US, ultrasound; AUC, Area under the curve.

Among individual ultrasound features, peripheral vascularization had the highest AUC (0.703, 95% CI: 0.644 - 0.757, *p* < 0.001), followed by microcalcification (AUC = 0.663, 95% CI: 0.603 - 0.719, *p* < 0.001), which demonstrated high specificity (86.6%, 95% CI: 78.2% - 92.7%) but low sensitivity (45.9%, 95% CI: 38.3% - 53.7%). Focal hyperechogenicity and round shape yielded lower diagnostic accuracy (AUC = 0.578 and 0.585, respectively), with the cystic degeneration showing the lowest overall performance (AUC = 0.542, 95% CI: 0.481 - 0.603, *p* = 0.03) (**Table 2**, **Figure 3**).

### Diagnostic performance of combining Node-RADS and other features

**Figure 4:**
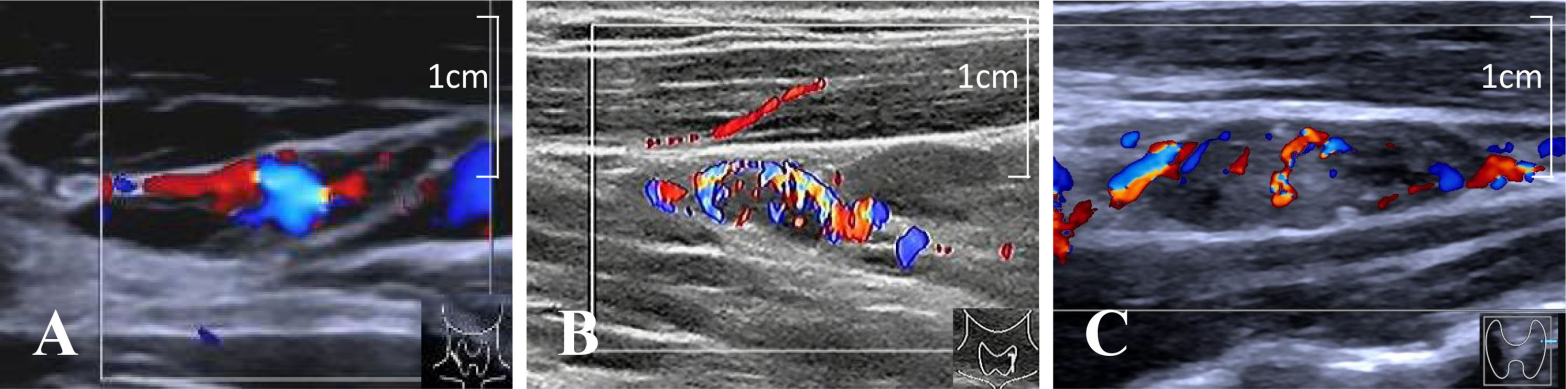
Representative vascular patterns of cervical LNs, including hilar **(A)**, peripheral **(B)**, and mixed **(C)**. LN, lymph node.

**Figure 5:**
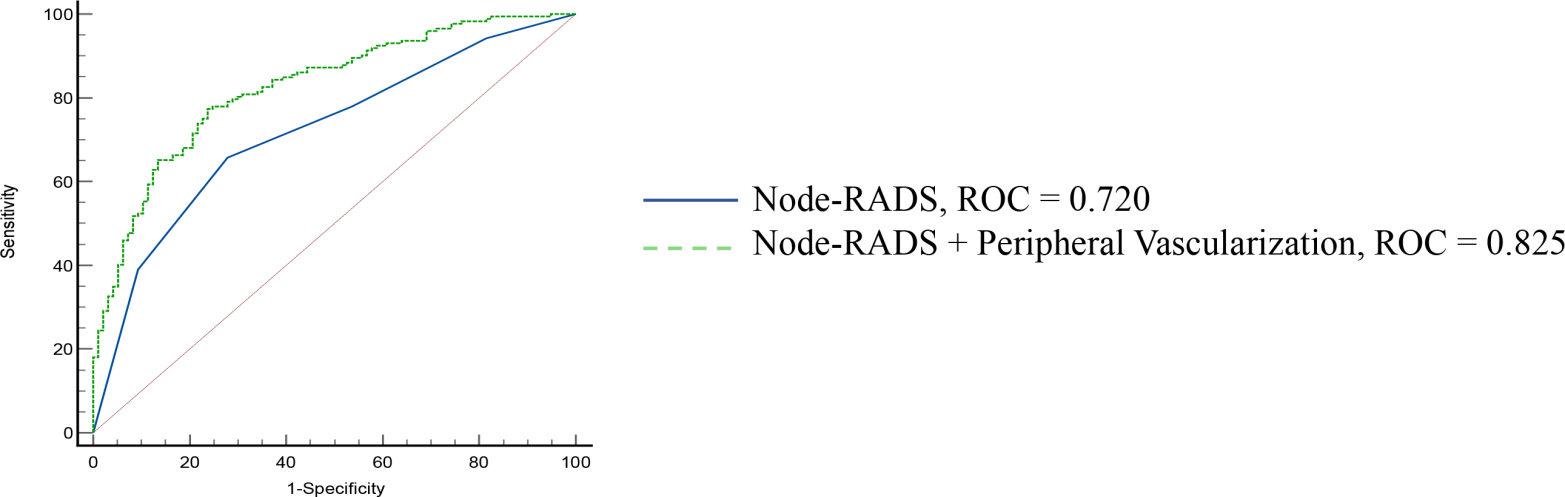
Diagnostic performance of Node-RADS and Node-RADS + peripheral vascularization. Node-RADS, Node Reporting and Data System; AUC, Area under the curve.

In multivariate logistic regression, both Node-RADS and peripheral vascularization were identified as independent predictors of cervical LN metastasis. The representative vascular patterns of cervical LNs were shown in **Figure 4**. The combined model incorporating these two variables significantly improved diagnostic performance (AUC = 0.825, 95% CI: 0.776–0.867, *p* < 0.0001), compared to Node-RADS alone (AUC = 0.720, 95% CI: 0.662–0.773, *p* < 0.0001) (*p* < 0.01) (**Table 3**, **Figure 5**). The result of 10-fold cross-validation procedure yielded a mean AUC of 0.814 (standard deviation: ± 0.039), thereby confirming the model’s robust discriminative performance.

**Table 3:** Results of univariable and multivariable logistic regression.

Characteristics	Univariable logistic regression		Multivariable logistic regression	
	*p*	OR (95% CI)	*p*	OR (95% CI)
Sex	0.50	1.21 (0.69, 2.13)		
Age	0.04	0.98 (0.96, 1.00)	0.08	0.98 (0.96, 1.00)
Cortical thickness (mm)	0.01	1.14 (1.04, 1.25)	0.68	0.98 (0.87, 1.10)
Node-RADS (Grade 1/2/34/5)	< 0.01		< 0.01	
	< 0.01	0.08 (0.03, 0.21)	< 0.01	0.09 (0.03, 0.30)
	< 0.01	0.14 (0.06, 0.33)	< 0.01	0.20 (0.07, 0.54)
	< 0.01	0.11 (0.05, 0.28)	< 0.01	0.12 (0.04, 0.34)
	0.02	0.34 (0.14, 0.83)	0.08	0.43 (0.16, 1.13)
Peripheral vascularization (absent/present/mixed)	< 0.01		< 0.01	
	< 0.01	0.17 (0.08, 0.39)	< 0.01	0.24 (0.09, 0.62)
	0.12	3.04 (0.76, 12.18)	0.11	3.61 (0.74, 17.77)
Vascular quantity on CDFI (G0/G1/G2/G3)	< 0.01		0.27	
Characteristics	< 0.01	0.17 (0.07, 0.41)	0.92	1.07 (0.32, 3.51)
	0.03	0.40 (0.18, 0.91)	0.43	1.57 (0.52, 4.77)
	0.77	0.85 (0.28, 2.55)	0.13	3.02 (0.71, 12.86)

Node-RADS, Node Reporting and Data System; CDFI, Color Doppler flow imaging; OR, odds ratio; CI, confidence interval.

## Discussion

While Node-RADS was originally developed for CT/MRI, its systematic adaptation and validation for US in the evaluation of cervical lymph nodes in PTC remains largely unexplored. As a primary study, our study not only confirms the feasibility of applying the Node-RADS framework in US, where its core criteria are fully applicable, but also moves beyond mere translation. We developed and validated a novel combined diagnostic model that integrates Node-RADS with the sonographic feature of peripheral vascularity, which significantly improved diagnostic performance (AUC increased from 0.720 to 0.825). Notably, we found a strong correlation between increasing Node-RADS scores and malignancy rates, with scores of 4 and 5 associated with a 71.9% and 88.2% malignancy risk, respectively. This work provides a critical foundation for and is part of an ongoing research program aimed at establishing a refined, US-specific scoring system for lymph node assessment in PTC.

The diagnostic accuracy of Node-RADS (AUC = 0.720, 95% CI: 0.662–0.773, *p* < 0.01) exceeded that of commonly used US features such as peripheral vascularity (AUC = 0.703, 95% CI: 0.644 - 0.757, *p* < 0.001), microcalcification (AUC = 0.663, 95% CI: 0.603 - 0.719, *p* < 0.001), and round shape (AUC = 0.585, 95% CI: 0.603 - 0.719, *p* = 0.01). This supported the premise that a composite scoring system integrating multiple morphologic criteria could outperform single-feature evaluations. Among the individual features, peripheral vascularity showed the highest AUC, consistent with previous studies reporting neovascularization as a hallmark of nodal metastasis (26, 28). However, features such as degeneration, although highly specific (92.8%, 95% CI: 85.7% - 97.0%), demonstrated low sensitivity (15.7%, 95% CI: 10.6% - 22.0%), limiting their standalone diagnostic value.

According to previous studies, the Node-RADS system has shown promising results in evaluating lymph node metastasis across various malignancies, including prostate, breast, lung, colon, and gastric cancers, using CT and MRI (29–32). However, its performance in distinguishing benign from malignant cervical lymph nodes by US in PTC patients was relatively modest, with an AUC of 0.720 (95% CI: 0.662–0.773, *p* < 0.01). A similar result was reported by Yu et al. by evaluating the diagnosis performance of Node-RADS using CT images to predict metastatic LNs in patients with PTC, with an AUC of 0.602 (5). This might be attributed to the smaller size of metastatic lymph nodes in PTC, which limits the ability to observe internal structural details. Additionally, the retrospective nature of this study might have introduced inaccuracies in assessing internal lymph node characteristics.

Importantly, our multivariate analysis revealed that both Node-RADS and peripheral vascularity were independent predictors of nodal metastasis. The combined diagnostic model (Node-RADS + peripheral vascularity) significantly improved diagnostic performance (AUC = 0.825, 95% CI: 0.776–0.867, *p* < 0.001), underscoring the complementary role of vascular features in enhancing malignancy prediction. The improvement in AUC is not only statistically significant but also suggests a clinically meaningful enhancement in risk stratification. This could potentially refine clinical pathways by more accurately identifying patients who would benefit directly from surgical intervention versus those for whom a confirmatory FNA remains the most prudent step. This performance was comparable to the risk stratification model for metastatic LN of PTC using US images developed by Ni et al. (AUC = 0.827) (33). These findings aligned with prior work suggesting that vascular flow patterns, particularly peripheral or chaotic vascularization, add incremental value to grayscale-based assessments (34, 35). The biological plausibility of this observation is supported by histopathologic studies. Specifically, peripheral vascularity on Doppler US has been shown to correlate with significantly increased microvessel density in the subcapsular sinus of metastatic lymph nodes (36). The underlying mechanism can be explained by the process of nodal metastasis: in the early stages, tumor cells arrive via lymphatic vessels and initially reside in the subcapsular sinus. These cells secrete pro-angiogenic factors such as VEGF, which is significantly overexpressed in metastatic lymph nodes of PTC (37), thereby inducing neoangiogenesis and resulting in the observed peripheral vascularization. As the tumor progresses, cells infiltrate deeper into the cortex and medulla, disrupting the normal nodal architecture and leading to more diffuse vascularization (38–41). This established pathologic sequence underscores the importance of peripheral vascularity as a critical indicator for assessing lymph node metastasis in PTC patients.

Our findings demonstrated that Node-RADS was a reproducible tool, with a substantial interreader agreement (*κ* = 0.67, 95% CI: 0.60–0.73), which suggested Node-RADS could facilitate more consistent interpretation among radiologists with varying experience levels. Previous studies have highlighted variability in LN characterization using traditional ultrasound criteria (7, 9); thus, standardized reporting systems such as Node-RADS might bridge the gap between subjectivity and reproducibility in clinical practice.

Our study had several limitations. First, this study is a single-center retrospective analysis, and the inclusion of only sonographically suspicious lymph nodes may introduce spectrum bias, potentially overestimating diagnostic performance. Additionally, the duration of clinical follow-up for benign lymph nodes was relatively limited. Also, the inter-scanner variability could affect the echogenicity and vascularity assessment. Therefore, the generalizability of our findings to unselected populations requires further validation through our ongoing prospective multi-center studies utilizing the same equipment and a standardized image archiving protocol with long-term follow-up. Second, despite rigorous reader training and blinding, subjective interpretation of ultrasound features might still influence Node-RADS scoring. Third, pathologic confirmation was not uniform, with some cases relying solely on cytology, which might carry limitations in diagnostic certainty. However, the high concordance rate of 92.2% (107/116) between FNA and subsequent surgical excision, in accordance with the literature (26, 27), supports the reliability of FNA as a precise technique, and the limitation can be overlooked.

In conclusion, our study is an exploratory, preliminary validation of Node-RADS as an effective and reproducible scoring system for US evaluation of cervical LNs in PTC patients. Its diagnostic performance surpassed that of individual ultrasound features, and combination with peripheral vascularity further enhanced accuracy. Future studies with larger and more diverse populations are warranted to refine the scoring criteria and validate its application across different clinical settings and disease types.

## Data Availability

The raw data supporting the conclusions of this article will be made available by the authors, without undue reservation.
